# Contrasting Photo-Switching Rates in Azobenzene Derivatives: How the Nature of the Substituent Plays a Role

**DOI:** 10.3390/polym12051019

**Published:** 2020-04-30

**Authors:** Domenico Pirone, Nuno A. G. Bandeira, Bartosz Tylkowski, Emily Boswell, Regine Labeque, Ricard Garcia Valls, Marta Giamberini

**Affiliations:** 1 Department of Chemical Engineering (DEQ), Rovira i Virgili University, Av. Països Catalans 26, 43007 Tarragona, Spain; domenico.pirone@estudiants.urv.cat (D.P.); ricard.garcia@urv.cat (R.G.V.); 2 Procter & Gamble Services Company n.v., Temselaan 100, 1853 Strombeek-Bever, Belgium; labeque.r@pg.com; 3 BioISI—Biosystems & Integrative Sciences Institute; C8, Faculdade de Ciências, Universidade de Lisboa Campo Grande, 1749-016 Lisboa, Portugal; nuno.bandeira@ciencias.ulisboa.pt; 4 Eurecat, Centre Tecnològic de Catalunya, C/Marcel-lí Domingo, 43007 Tarragona, Spain; bartosz.tylkowski@ctqc.org; 5 The Procter and Gamble Company, 8611 Beckett Rd, West Chester Township, Cincinnati, OH 45069, USA; boswell.ec@pg.com

**Keywords:** photoswitching, azoaryls, azobenzene, UV-Visible, TD-DFT, kinetics, photoisomerization

## Abstract

A molecular design approach was used to create asymmetrical visible light-triggered azo-derivatives that can be good candidates for polymer functionalization. The specific electron–donor substituted molecules were characterized and studied by means of NMR analyses and UV-visible spectroscopy, comparing the results with Time Dependent Density Functional (TD-DFT) calculations. A slow rate of isomerization (k_i_ = 1.5 × 10^−4^ s^−1^) was discovered for 4-((2-hydroxy-5methylphenyl) diazenyl)-3-methoxybenzoic acid (AZO1). By methylating this moiety, it was possible to unlock the isomerization mechanism for the second molecule, methyl 3-methoxy-4-((2-methoxy-5-methylphenyl) diazenyl)benzoate (AZO2), reaching promising isomerization rates with visible light irradiation in different solvents. It was discovered that this rate was heightened by one order of magnitude (k_i_ = 3.1 × 10^−3^ s^−1^) for AZO2. A computational analysis using density functional (DFT/PBE0) and wavefunction (QD-NEVPT2) methodologies provided insight into the photodynamics of these systems. Both molecules require excitation to the second (S_2_) excited state situated in the visible region to initiate the isomerization. Two classic mechanisms were considered, namely rotation and inversion, with the former being energetically more favorable. These azo-derivatives show potential that paves the way for future applications as building blocks of functional polymers. Likewise, they could be really effective for the modification of existing commercial polymers, thus transferring their stimuli responsive properties to polymeric bulky structures, converting them into smart materials.

## 1. Introduction

**Figure 1:**
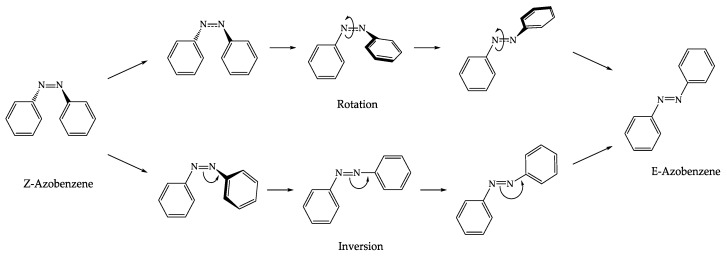
Rotation and inversion thermal relaxation mechanisms for unsubstituted azobenzene.

A great attention during the past decades has been addressed to photochromic molecules in the field of materials science. These organic compounds can be included in many materials, changing the properties of the system when irradiated with light of the appropriate wavelength. The change can affect characteristics like: electronic or ionic conductivity, fluorescence, magnetism, shape, and conformation. The most studied photochromic molecules are azobenzenes [1] which have been widely investigated as promising photo-switchers [2,3,4,5,6] due to their simple preparation procedures, good processability, as well as high thermal and chemical stability [7]. However, the main interesting characteristic of azobenzene molecules is their reversible isomerization of the double bond, between the thermally stable *E* (trans) configuration and the meta-stable *Z* (cis) one [8,9,10,11,12]. This process drives to change the geometry of the molecule. Structurally, while the *E* form is approximately planar, the *Z*-isomer is three-dimensional and has a ground state energy nearly 0.6 eV higher than the *E* one [13,14,15]. This interconversion between the two isomers makes the azobenzene the most used organic chromophore for technical applications, including optical waveguides and shutters [16], optical memories [17], photo-active artificial muscles [3,4,5], photo triggered carriers and membranes [18,19,20,21,22], etc. A photostationary state (PSS) will be achieved by a solution containing an azo derivate when irradiated by electromagnetic radiation of the right intensity, with a steady-state *E–Z* composition based on the opposing effects of photoisomerization and thermal relaxation back into the *E* state. The composition of the sample also depends on the irradiation wavelength, intensity, solvent-solute interactions and temperature. Moreover, the optical properties, the response time of the isomerization, and the spectroscopic properties of an azo molecule can be fine-tuned by attaching to the phenyl rings different chemical groups [23]. For example, it is usually accepted that *Z–E* thermal relaxation may take place by means of two pathways that can be competitive (Figure 1): rotation, or inversion of one of the nitrogen atoms [24,25]. Depending on the properties of the substituents bonded to one or both phenyl rings, and on the polarity of the reaction medium, one of these mechanisms of relaxation may be favored over the other [26]. 

Rau has divided the azobenzenes into three spectroscopic classes based on their substitution patterns on the phenyl rings: azobenzene-type molecules, aminoazobenzene-type molecules, and pseudo-stilbenes, respectively [1]. The pseudo-stilbenes have presented the most efficient photo-response. This behavior must be linked to a mixed photo-stationary state where the *Z* and *E* form are being constantly interconverted, caused by a shifted and overlapped absorption spectrum of the two isomers. Consequently, for these compounds, both the forward and reverse isomerization can be induced by a single wavelength of light in the visible region. On the other hand, the azobenzene-type molecules can isomerize very slowly from the *Z* configuration back into the *E* configuration when bulky substituents are introduced to delay the thermal relaxation [27,28]. Recently, the studies provided by Jerca et al. revealed that, using the proper solvent, it is possible to yield stable *Z*-isomers. This behavior can be enhanced by the nature and position of substituents on the aromatic rings [29]. This last feature of the azobenzene-type molecules is a very promising tool that allows fine-tuning of the optical response. Understanding the isomerization kinetics of different substituted azo-derivatives in solution is of great importance in order to gain control over the response time to light irradiation and design photo responsive materials for precise applications. For instance, these azo systems can be used as moieties to bring stimuli-responsive polymers into a broad range of already existing commercial polymeric families, thus transferring their stimuli responsive properties to polymeric bulky structures with the aim of enhancing materials performances. 

**Scheme 1:**
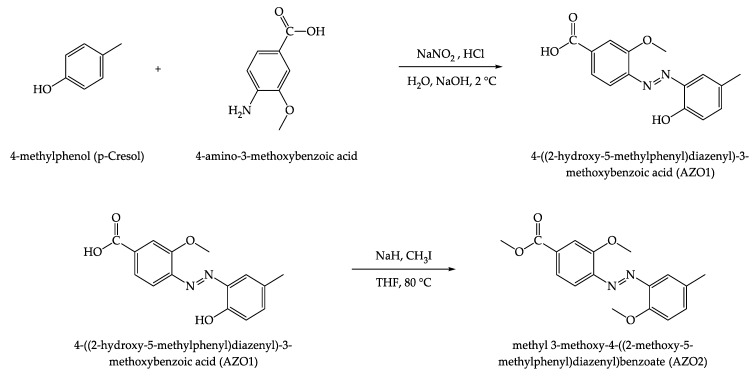
Synthetic paths of 4-((2-hydroxy-5methylphenyl) diazenyl)-3-methoxybenzoic acid (AZO1) and methyl-3-methoxy-4-((2-methoxy-5-methylphenyl) diazinyl) benzoate (AZO2).

The idea of our work was to design, synthesize, and study novel asymmetrical azobenzene compounds with a precise substitutional pattern, bearing one reactive site. The specific properties derived from their substitutional pattern and the single reactive site could make them good candidates for the chemical modification of commercial polymers. The main goal was to create a proper photosensitive active group that, by means of its reactive site, could be grafted to a wide range of polymers, thus giving them stimuli-responsive properties. We focus our attention on the study of the *E–Z–E* isomerization kinetics of two related novel azo-derivatives (shown in Scheme 1). To the best of our knowledge, the synthesis of these two compounds has not been reported in the literature. These systems were well-designed to achieve an isomerization when irradiated by visible light and a thermal back isomerization when heated above room temperature. We investigated the conversion process in silico with a static approach (time dependent DFT) and the photodynamics of isomer conversion through a multi-configurational wavefunction methodology (QD-NEVPT2) in order to have a complete understanding of the properties of such systems. In the course of our investigation, some unusual and unexpected photochemistry of these asymmetrical ortho substituted derivatives were discovered. These findings can provide new insights regarding azobenzene isomerization as well as the possibility to reveal new applications for a well-known organic molecule. For instance, through a reactive site such as a carboxylic group, these photoactive moieties can be conveniently grafted to several classes of commercial polymers, such as polyvinyl alcohols (PVAs) and polyepichlorohydrin (PECH), thus conferring upon them light responsiveness [19]. In this way, commercial polymers could behave as novel smart materials.

## 2. Materials and Methods 

### 2.1. Materials

Briefly, 4-Amino-3-methoxybenzoic acid (TCI, Tokyo Chemical Industries, ≥99% pure, TCI Europe, Boereveldseweg 6 - Haven 1063, 2070 Zwijndrecht, Belgium), 4-methylphenol (p-Cresol) (TCI, Tokyo Chemical Industries, ≥98% pure), methyl iodide (CH_3_I, 141.94 g/mol), sodium hydride (NaH, 50% dispersion in mineral oil), sodium hydroxide (NaOH) and sodium nitrite (NaNO_2_) were purchased from Merck Spain (Merk Spain, Calle de María de Molina, 40, 28006 Madrid, Spain) and used without further purifications. Tetrahydrofuran (THF) was pre-dried over calcium hydride and distilled from sodium benzophenone ketyl, according to standard procedures. Dimethyl sulfoxide (DMSO), N,N-dimethylformamide (DMF), and dioxane, spectrophotometric grade, were purchased from Merck and used as received.

### 2.2. Characterization

Subsequently, ^1^H NMR and ^13^C NMR spectra were recorded at 400 and 100.4 MHz, respectively, on a Varian Gemini 400 spectrometer (Varian, Inc. 3120 Hansen Way, Palo Alto, CA, USA) with proton noise decoupling for ^13^C NMR. The chemical shifts were given in parts per million from TMS (Tetramethylsilane) in ^1^H NMR spectra, while the central peak of the solvent was taken as the reference in the case of ^13^C NMR spectra. The ^13^C NMR spectra of the polymers were recorded at 30 °C, with a flip angle of 45°, and the number of transients ranged from 20,000 to 40,000 with 10%–20% (w/v) sample solutions in deuterated dimethyl sulfoxide (DMSO-d6). A pulse delay time of 5 s for the ^1^H NMR spectrum was used. Quantitative ^13^C NMR spectra were performed using deuterated dimethyl sulfoxide (DMSO-d6) at 30 °C with a pulse delay time of 5 s. Photoisomerization was investigated using a UV-1800 Shimadzu Spectrophotometer (SHIMADZU EUROPA GmbH, Albert-Hahn-Strasse 6-10, 47269 Duisburg, Germany) with a scanning rate of 3000 to 2 nm/min. The kinetics of photoisomerization and thermal relaxation were followed by the same instrument. The samples for UV-visible spectroscopy were prepared via dilution of the sample into adequate solvent (DMSO, DMF, Dioxane) to give a final concentration of 7.4 × 10^−5^ M in red light room (λ > 600 nm), to assure that they were predominantly in *E* form. After maintaining the azo dye solutions in the dark overnight, the spectra were taken in a UV-grade quartz cell with optical beam path of 1 cm before and after irradiation with a desk lamp equipped with a 11 W A E27 ID60 LED bulb (Jedi Lighting iDual bulb, TAO UK, 4 Deanfield Court, Link 59 Business Park, Clitheroe, Lancs, UK). During the test the sample was placed at 2 cm from the light source. The optical signal output of white light was measured by an Ocean Optics USB2000 Miniature Fiber Optic Spectrometer (StellarNet, Inc., 14390 Carlson Circle, Tampa, FL, USA) in a dark room at room temperature, for more details the measured spectrum is shown in Figure S1 in the SI (3890 lux at 2 cm and 1030 lux at 8 cm, from the source, respectively). The solution was analyzed after 5 min irradiation. The process was repeated several times since the photostationary state (PPS) was observed. The *Z*–*E* thermal relaxation of solutions was studied after irradiating the samples reaching the photostationary state with the iDual lamp and then heating them in a TCC-100 Thermoelectrically Temperature Controlled cell holder (Aragonesses 13, Polig. Industrial 28100, Alcobendas, Madrid, Spain) for the analysis at different temperatures. The instruments collected the spectra each 2 min for 30 min. The temperatures used for the experiment were: 30, 40, 50, and 60 °C. Background was performed on each of the solvents used both before and after light irradiation and confirmed that the solvents absorption spectrum did not affect the region between 250 and 600 nm.

### 2.3. Synthesis of 4-((2-hydroxy-5-methylphenyl) diazenyl)-3-methoxybenzoic acid (AZO1) and Methyl-3-methoxy-4-((2-methoxy-5-methylphenyl)diazinyl)benzoate (AZO2)

For the synthesis of 4-((2-hydroxy-5-methylphenyl) diazenyl)-3-methoxybenzoic acid (AZO1), 12 mmol of 4-Amino-3-methoxybenzoic acid, 80 mL of water, and 7 mL of 35% hydrochloric acid were introduced in a 100 mL beaker and mildly heated at 40 °C to get the amine chlorohydrate (Scheme 1). The resulting suspension was then cooled to ≈ 0 °C in an ice bath. Meanwhile, 1.35 g (19.6 mmol) sodium nitrite were dissolved in 2.8 mL of water and added dropwise to the first suspension to obtain the corresponding diazonium salt (solution A). Solution B was prepared by dissolving 1.616 g (15 mmol) p-Cresol in 14 mL of water with 2.10 g (52.5 mmol) of sodium hydroxide. Solution B was then carefully added dropwise to solution A at 2–3 °C. A dark red precipitate was obtained. The crude product was then re-dissolved in 200 mL of a 10% aqueous solution of sodium hydroxide, the solution was diluted with 800 mL of water and then 20 mL 37% hydrochloric acid were added carefully. The product precipitated as a pure red solid which was then filtered and dried in oven at 45°C; yield 58%, m.p.: 262.7 °C (deg.)

For the methylation of the hydroxyl groups of the previous molecule and the production of methyl-3-methoxy-4-((2-methoxy-5-methylphenyl) diazinyl) benzoate (AZO2), to 7.4 mmol of AZO1 in 50 mL of dry THF, 0.83 g of NaH were added (60% dispersion in mineral oil) followed by 1.73 mL (27 mmol) of methyl iodide. The mixture was stirred at 80 °C for 24 h. The solvent was evaporated with a rotavapor and the product was recrystallized from 2-propanol, obtaining an orange fine powder. Yield: 49%, m.p.: 138.4 °C.

The spectral characterization of both compounds is summarized below. 

AZO1: ^1^H NMR (DMSO-d6) δ: 12.3 (s, 1H, OH), 7.8 (d, J = 8.3 Hz, 1H), 7.7 (d, J = 1.5 Hz, 1H), 7.6 (dd, J = 3.8, 1.2 Hz, 1H), 7.6 (d, J = 1.6 Hz, 1H), 7.3 (m, 1H), 6.9 (d, J = 8.4 Hz, 1H), 4.0 (s, 3H), 2.3 (s, 3H).^13^C NMR (DMSO-d6) δ: 167.0 (s); 155.9 (s); 151.9 (s); 142.1 (s); 134.6 (s); 129.3 (s); 118.5 (s); 114.1 (s); 56.7 (s); 20.3 (s).

AZO2: ^1^H NMR (DMSO-d6) δ: 7.7 (d, J = 1.3 Hz, 1H), 7.5 (dd, J = 8.2, 1.4 Hz, 1H), 7.4 (d, J = 8.2 Hz, 1H), 7.3 (dd, J = 8.4, 1.9 Hz, 1H), 7.2 (d, J = 1.8 Hz, 1H), 7.1 (d, J = 8.5 Hz, 1H), 4.0 (s, 3H), 3.9 (s, 3H), 2.3 (s, 3H). ^13^C NMR (DMSO-d6) δ: 169.5 (s); 156.4 (s); 155.2 (s); 144.9 (s); 142.6 (s); 142.1 (s); 133.4 (s); 129.8 (s); 121.8 (s); 117.0 (s); 115.8 (s); 113.9 (s); 113.7 (s); 56.5 (s); 56.2 (s); 20.5 (s).

### 2.4. Computational Methods

Density functional calculations were performed using the ADF [30] 2017 103 modelling suite, employing the Perdew, Burke, and Ernzerhof hybrid functional (PBE0) [31,32,33]. The basis sets consisted of all electron triple zeta Slater type orbitals with one polarization function (TZP). The COnductor like Screening MOdel (COSMO) dielectric continuum solvation scheme was employed in the calculations with default appropriate parameters for dimethylformamide, dimethyl sulfoxide and dichloromethane [34]. Geometry optimizations did not involve any symmetry constraints. Time dependent (TD-PBE0) runs for the calculation of UV-visible transitions required 60 singlet-singlet excitations [35].

Additional multiconfigurational self-consistent field calculations [36] were performed using the ORCA 4.0.1 software package [37,38]. The PES scans were carried out by relaxing the geometries in vacuo at the state average (six singlets) CASSCF (4,3) level to a minimum at every scan point. At each of the scan points the wavefunction subsequently underwent a perturbation through the Quasi-Degenerate N-Electron Valence Perturbation Theory (QD-NEVPT2) approach [39,40] in the Nakano formulation [41]. The Ahlrichs def2-TZVP contracted Gaussian type basis set [42] was employed for every element making use of the resolution of the identity (RI) Coulomb integral approximation scheme with the def2-TZVP/C correlation density fitting basis set [43]. Using the state average CASSCF wavefunction, a non-SCF CI run was performed for the ground (S_0_) and second excited (S_2_) states which then underwent a density matrix analysis using the natural bond orbital scheme (NBO 6 program [44]). The natural localized molecular orbitals were generated, and their bond orders computed using the procedure devised by Schleyer et al. [45].

## 3. Results & Discussions

### 3.1. Molecular Design of the Azo-derivatives:

Azo compounds are usually synthesized with procedures like: azo coupling reaction (coupling of diazonium salts with activated aromatic compounds), Mills reaction (reaction between aromatic nitroso derivatives and anilines) and Wallach reaction (transformation of azoxybenzenes into 4-hydroxy substituted azo-derivatives in acid media) [46]. They are mostly produced with symmetric structures that have two potential reactive sites that offer the possibility to combine them with other monomers. In this way, polymers capable to react when irradiated by an electromagnetic irradiation can be produced. They are typically sensitive to UV light irradiation and they can have unstable *Z* form that can convert back to the more stable *E* form within picoseconds. 

A preliminary study on the possible substitution pattern for the novel azobenzene derivatives synthesized in this work was performed. The objective was to achieve a well-designed asymmetric structure to:

- Produce a molecule with only one reactive site for further modifications and be used as a side-chain group.
- Move the excitation wavelength of the *E-Z* isomerization from the UV region of the absorbance spectrum to the visible light one.
- Enhance the stability of the *Z* form once obtained after irradiation with white light.

To successfully synthesize the first compound AZO1, an azo coupling reaction procedure was chosen with p-Cresol and 4-amino-3-methoxybenzoic acid as precursors. In this specific electrophilic aromatic substitution reaction, a phenol with a methyl group in *para* position was selected as the activated arene. The substitution occurs stereotypically in *para* to the activating group when a bulky substituent is selected as the attacking species. However, *ortho* substitution takes place when the *para* position is already occupied by another substituent. The 4-amino-3-methoxybenzoic acid was chosen to give the molecule the desired asymmetry and a reactive site for further modifications. In addition, with the presence of an electron donor group on the aromatic ring, like the methoxy one, we aimed to move the isomerization wavelength to the visible region of the absorption spectrum to trigger the isomerization with white light irradiation [47]. Lastly, it was reported that the methyl group could enhance the stability of the *Z*-isomer prolonging its half-life in the darkness [48].

The conditions of the reaction were carefully controlled both in pH and temperature due to the instability of the diazonium salts formed during the process. A mildly acidic or neutral solution is usually needed when an aromatic amine is involved in this reaction: nevertheless, if the solution is too acidic, the concentration of free amine becomes too small and the reaction does not occur. On the other hand, the phenol is usually not sufficiently active for the reaction, but, the more reactive phenoxide ion is a better initiator to generate the target azobenzene, when slightly alkaline conditions are present. Nonetheless, neither amines nor phenols can react in moderately alkaline solution because the diazonium ions are converted into the corresponding diazo hydroxide [49].

From the account given by Bandara et al. [50] regarding a specific set of azobenzene derivatives, the hydroxyl groups can hinder the isomerization by forming hydrogen bonds with the azo group, or other substituent on the opposite ring. We therefore envisaged to apply this same idea for the present work. The AZO1 compound was thus methylated to give AZO2 (Scheme 1). This modification generates a structure with two electron donor groups in *ortho* position on both the phenyl rings, changing the conjugation of the electrons on the –N=N– double bound. This modification was needed given the small isomerization conversion registered for AZO1 *E-*isomer when irradiated by white light, as shown in the following sections.

The novel azobenzene compounds were obtained with reasonably good yields and purities. Their structures were characterized by ^1^H–NMR, ^13^C–NMR, and UV-Vis spectroscopy. 

### 3.2. H–NMR and ^13^C–NMR Characterizations:

**Figure 2:**
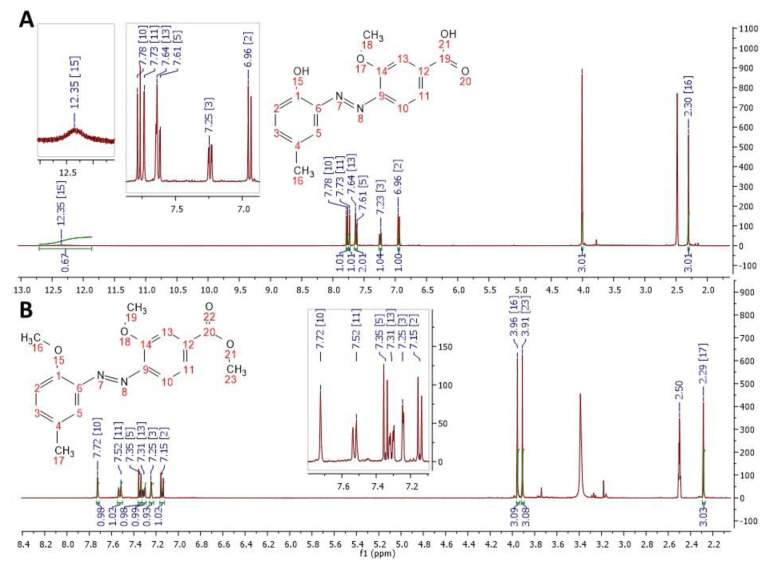
^1^H–NMR spectrum comparison of 4-((2-hydroxy-5-methylphenyl)diazenyl)-3-methoxybenzoic acid (AZO1) and methyl-3-methoxy-4-((2-methoxy-5-methylphenyl)diazinyl) benzoate (AZO2): (**A**) and (**B**), respectively.

In Figure 2 ^1^H–NMR spectra of AZO1 and AZO2 molecules dissolved in deuterated DMSO are shown. The proton spectra exhibited some similarities. The peaks corresponding to the aromatic hydrogens are highlighted in the insets showing the δ region between 7.0 ppm and 8.0 ppm. The peaks related to the hydrogen atoms of the methoxy and methyl groups are present in the region from 2.0 to 4.0 ppm. A peak corresponding to the –OH of the carboxylic group is visible at 12.18 ppm (Figure 2A). After the conversion to methyl ester, this peak disappeared as it is evident in the Figure 2B. On the other hand, a peak corresponding to the methyl ester group is present at 3.91 ppm. All the peaks of the aromatic rings were shifted after the methylation, but their number remained unchanged as expected. The small peak at 3.33 ppm is characteristic of the residual water in d-DMSO. From these evidences, one can conclude that the methylation of the AZO1 molecule was successfully accomplished. AZO2 spectrum in d-DMSO at 30 °C at the PSS is shown in Supplementary Figure S7, and for AZO1, no changes were measured by NMR analyses after irradiation.

The ^13^C–NMR chemical shifts of the molecules AZO1 and AZO2 are given in Table 1. From the data shown, it is evident that two new signals appear after the methylation of AZO1. They can be related to the two additional carbon atoms present in the AZO2 structure after AZO1 methylation.

### 3.3. UV/Vis Spectra Interpretation:

The UV–visible absorption spectra of the azo-derivatives exhibit two characteristic absorption bands. The first characteristic absorption band is present around 330–320 nm, while the second absorption band is present around 412–325 nm.

Comparing the absorption spectra, it is evident that the methoxy groups attached on both the phenyl rings of AZO2 are blue shifting the λ_1_ of 26 nm in DMF (Table 2). This can be explained by the lower conjugation effect that they induce on the –N=N– bond electrons. 

**Figure 3:**
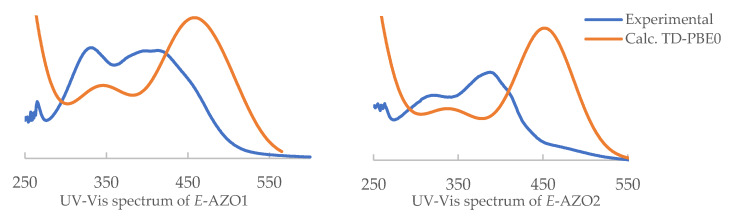
Measured and calculated absorption spectra of *E*-AZO1 (**left**) and *E*-AZO2 (**right**) in dimethylformamide.

**Figure 4:**
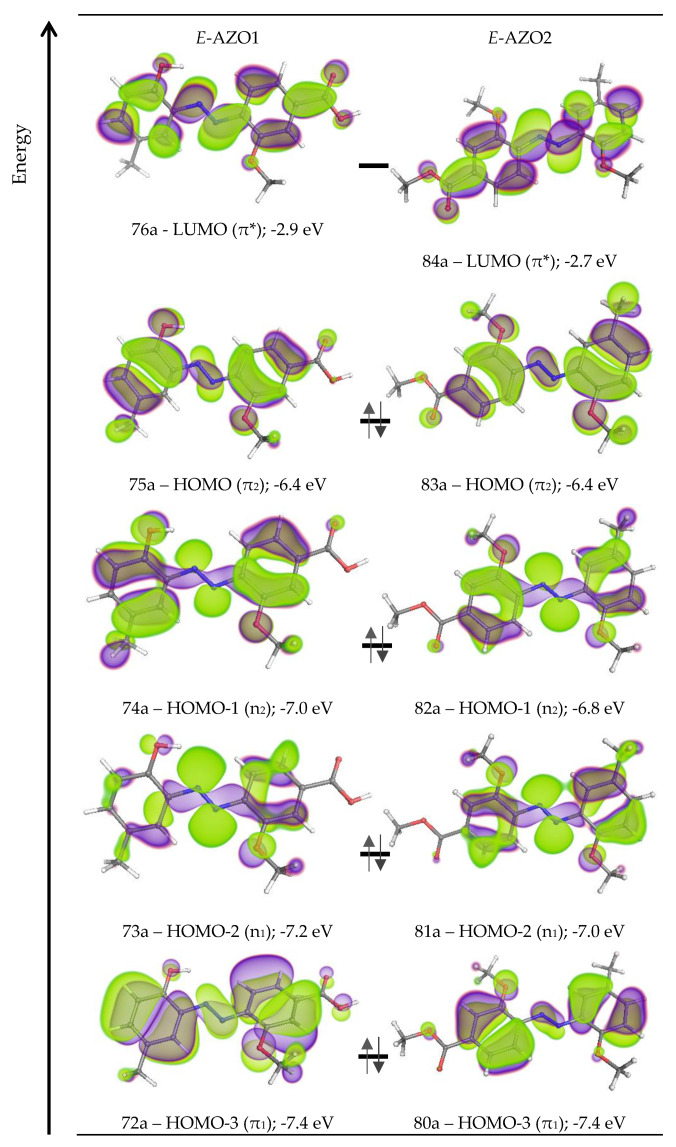
Kohn-Sham molecular orbitals relevant to the electronic transitions of *E*-AZO1 and *E*-AZO2 in the visible region.

To understand the optical properties of the two compounds, an in-silico analysis was carried out with the time dependent density functional (TD-DFT) approach. A DFT geometry optimisation (see computational methods) was performed for the ground state for both structural isomers of AZO1 and AZO2. The simulated electronic vertical transitions of both molecules are displayed on Figure 3 and these will occur in the frontier orbital region mapped out in Figure 4. The calculated bands (TD-PBE0) compare favourably well to the experimental ones, presenting a redshift in the region of 20–50 nm. The relative intensities are incorrectly described, likely due to solvent interaction effects.

The band maxima corresponding to the electronic excitations are listed in Table 3 and Table 4 for AZO1 and AZO2, respectively. The most intense transition in the visible region can be assigned to the second excited state for both *E*-AZO1 and *E*-AZO2, each showing oscillator strengths of 0.57 and 0.91 respectively. The conventional assignment of distinctly separate n→ π* and π→ π* transitions is not rigorously valid in the case of *E*-AZO1 since there is an admixture of both transitions in the first two bands.

The first and second electronic transitions of *E*-AZO1 correspond to two maxima calculated to be at 489 and 441 nm and have a majority of π_2_→ π* character. The other band with significant oscillator strength in this compound is due to a π_1_→ π* transition occurring at 338 nm.

**Figure 5:**
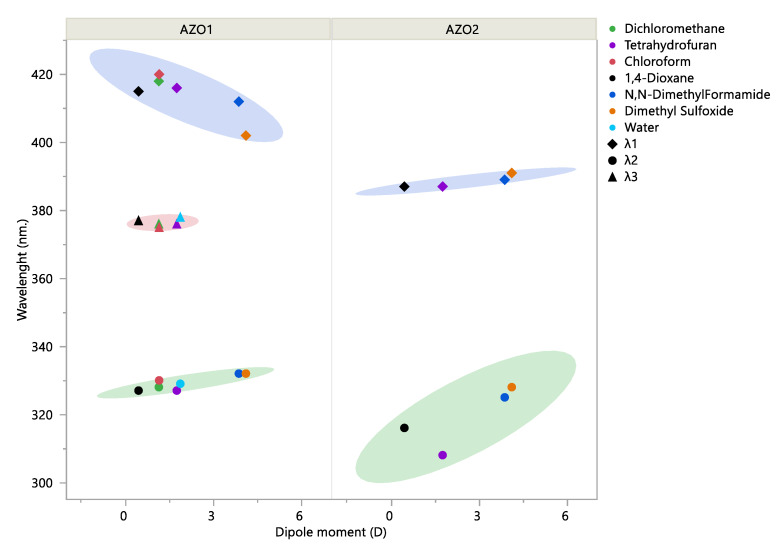
Band absorption maxima of AZO1/AZO2 correlated with solvent dipole moment (Debye).

The spectral features of *E*-AZO2 are altogether significantly different in that n→ π* transitions do not admix with the π→ π* type excitations. Since the former transitions are symmetry forbidden, they have a very low oscillator strength while the latter show up as very strong intensity bands. To broaden the study on the optical properties of the two moieties, the samples were dissolved in different solvents, according to the solubility of the different molecules. Figure 5 shows the dipole moments (in Debye) of each solvent compared to the wavelengths of the main peaks (λ_1_, λ_2_, λ_3_) of the absorption spectra of the azo-derivatives.

For AZO1 molecule, a broader range of solvents could be tested. The first peculiar aspect is that AZO1 main peak (λ_1_) is splitting its signal passing from a more polar solvent like dimethyl sulfoxide to a less polar one like dichloromethane. This can be ascribed to the different mechanisms of excitation followed by the electrons involved in the azo double bond when irradiated by light. In more polar solvents the signal linked to this excitation are overlapped (λ_1_), then their wavelength split (λ_1_ and λ_3_) being better resolved when the polarity of the solvent decreases. The second crucial aspect is highlighted by the hypsochromic shift of λ_1_ and the bathochromic shift of λ_2_ of AZO1 molecule in polar solvents. This can be related to the stabilization of the electronic cloud of AZO1, that is favoured by the presence of a less polar solvent like dioxane. This, in fact, can decrease the conjugation effect of the electrons of the azo double bond, shifting lambda to lower wavelengths. AZO2 λ_1_ is slightly influenced by the solvent polarity, a bathochromic shift is registered passing from a less polar solvent to a more polar one [51]. 

The UV-Vis spectrum was calculated for *E*-AZO1 in various implicit solvent dielectric media to determine how the effect that polarity shifts the visible band wavelengths. As can be seen from the values in Supplementary Table S4, the values of λ_1_ tend to increase when passing from vacuum to the solvated environment.

Neglecting dispersion interactions between solvent and solute represents the limiting case of solvation in non-polar media. However, the value of λ_1_ changed only a few for the used selection of solvents, with varying degree of polarity differences. The observed trend for DMSO is not reproduced by the calculation and in fact the value is slightly higher with respect to CH_2_Cl_2_ and DMF, but the change is so small that barely any significance can be inferred from it. The failure to account for the experimental trend is a sign that explicit interactions between the solute and the solvent, which are not considered at this level of theory, are essential to explain the subtle band shifts.

### 3.4. E–Z Photoisomerization

**Figure 6:**
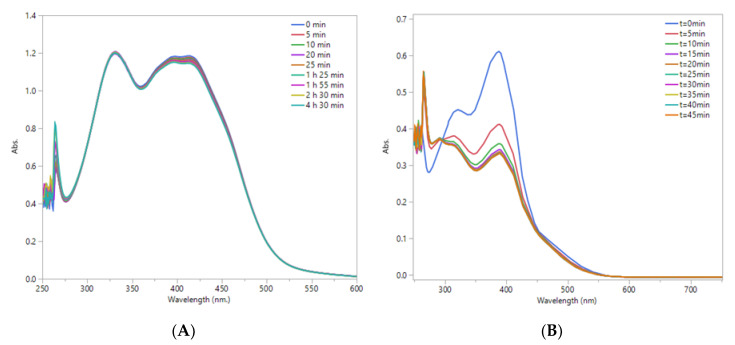
UV/Vis Absorption changes upon irradiation of visible light. From left to right, (**A**) AZO1 and (**B**) AZO2 absorption spectra.

The photoisomerization kinetics of azo-derivatives was established by monitoring the absorbance of S_2_ absorption band. Figure 6 shows the UV–Vis spectra of the AZO1 and AZO2 molecules after irradiation at different times. Under white light irradiation the intensity of the S_2_ absorption band gradually decreases with the irradiation time, while the S_4_ absorption band increases, until the photo-stationary state is reached. Both the solvent polarity and substituent nature influence the composition of the PSS. In fact, comparing the isomerization of both molecules, there are big differences that are easy to point out. First, only a slight change is visible in AZO1 absorption spectra (Figure 6A). The *E-Z* conversion rate is about 3.3 % after 4 h 30 min of irradiation compared to 45.7% of AZO2 after 50 min of irradiation. The kinetic constant of the isomerization for the two molecules differ by an order of magnitude (Table 2). 

Table 5 lists the photoisomerization rate constants calculated from UV–Vis experiments on AZO2 in different solvents at 303K. It can be observed that AZO2 k_i_ increases going from a less polar solvent like dioxane to a more polar one, such as DMSO. This evidence can be related to the photoisomerization mechanism. When rotation or inversion-assisted rotation pathway is preferred, an acceleration of the photoisomerization rate in polar solvents is noticed, due to the stabilization of the dipolar transition state. A change in the isomerization mechanism can be responsible for the lower percentage of *Z* fraction registered when AZO2 is dissolved in dioxane [51].

### 3.5. Z–E Thermal Relaxation

The kinetic *Z–E* thermal relaxation of AZO1 and AZO2 were investigated at several temperatures in order to determine its thermodynamic parameters. However, in the case of AZO1, thermal relaxation was impossible to study due to the hindered isomerization. A rough estimation of the kinetic constant relative to *Z-E* thermal relaxation of AZO1 was attempted from the data obtained in DMF: unfortunately, the obtained value is not reliable because it is falling within the experimental uncertainty of the instrument (for sake of explanation the tentative fitting is shown in the SI with a value R^2^ = 0.040). The rate of *Z–E* thermal relaxation of AZO2 molecule was monitored in the dark at the absorption maximum of its *E-*isomer. The thermal *Z* to *E* isomerization rate, summarized in Table 6, was determined for AZO2 at different temperatures and solvents. The process was found to be first order in all the studied solvents.

Calculation of the first-order rate constants at various temperatures allowed us to estimate thermodynamic activation parameters such as the activation energy (E_a_), activation enthalpy (ΔH^‡^), and activation entropy (ΔS^‡^) for thermal *Z* to *E* reaction using Arrhenius and Eyring equations (equations shown in Appendix A). We used the linear correlation between the enthalpy and the entropy of activation to determine the mechanism of AZO2 thermal relaxation. In all the solvents, the Arrhenius plots were linear, proving that there is no significant change in the reaction pathway over the examined temperature range. AZO2 compound has a positive enthalpy of activation and negative entropy for all the solvents used (see Table 7).

AZO2 in DMF has the highest ΔS^‡^ absolute value and the lowest ΔH^‡^, and therefore exhibits the fastest thermal relaxation [52,53]. This is consistent with the calculation that will be shown below. In order to understand the dynamic photochemical and thermal processes involved in the isomerisation of AZO1 and AZO2 a choice of appropriate computational strategy was paramount. The employment of density functional theory is unsuitable in this instance because there is no guarantee, that at every stage in the transformation within the specific excited state hypersurface, the electron wavefunction of the ground state remains singly determinantal. The best methodology is to adopt multi-configurational self-consistent field theory [36,54] with a posteriori perturbational corrections for a good description of dynamical electron correlation such as the NEVPT2 approach [40,55] (see Computational Methods).

Two conventional mechanisms usually adopted to describe the isomer change involve the either the twisting of the ∡(N=N–C) angle (inversion) or a rotation of the planes that make up the ∡(C-N=N–C) dihedral (rotation). Only the latter was found to exhibit more favourable energies for the photochemical conversion. This contradicts the hydrogen bonding inhibition scenario as presented by Bandara et al. [50], since rotation of the molecule around the –CNNC– dihedral planes does not necessarily require the termination of the intramolecular hydrogen bond with the adjacent hydroxyl group. An alternative explanation is given below to account for the slower rates of photo-switching.

The alternative twisting scenario may be found in the supporting information section. More elaborate reaction coordinates combining angular and dihedral changes have also been studied for unsubstituted azobenzene [56].

As seen from Table 3 and Table 4 the second excited state (S_2_) presents the highest oscillator strength for both AZO1 and AZO2: this will be the most efficient conversion pathway.

**Figure 7:**
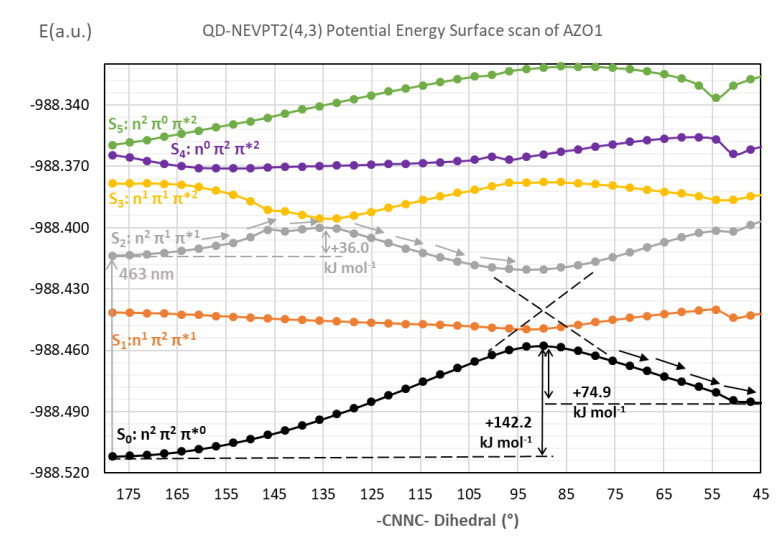
QD-NEVPT2(4,3)//SA6-CASSCF (4,3)/def2-TZVP potential energy surface scan of the six lowest lying singlet states of AZO1. The S_2_ and S_3_ have an avoided crossing at approximately ∡(CNNC) = 139° and some 8.6 kcal/mol above the S_2_ minimum.

**Figure 8:**
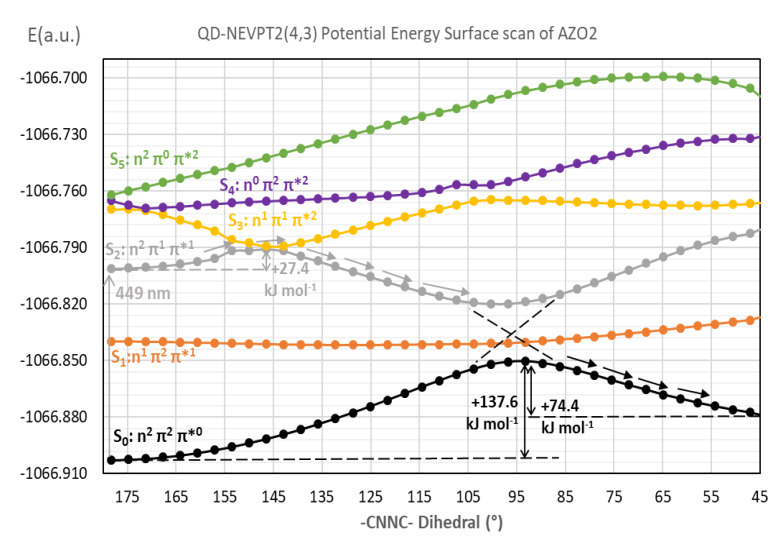
QD-NEVPT2(4,3)//SA6-CASSCF(4,3)/def2-TZVP potential energy surface scan of the six lowest lying singlet states of AZO2. S_2_ and S_3_ states cross at approximately ∡(CNNC) = 150°and 6.6 kcal/mol above the S_2_ minimum.

A relaxed potential energy surface (PES) scan of the –CNNC– dihedral at the QD-NEVPT2(4,3)//CASSCF (4,3)/def2-TZVP level of theory is presented in Figure 7 and in Figure 8 for AZO1 and AZO2, respectively. Therein the energies of each of the lowest six singlets were monitored throughout this reaction coordinate. The main composition of the states at the beginning of the process (*E*-isomer) is qualitatively described with different colours. The curves are qualitatively similar to what was previously reported for azobenzene [57].

The photochemical process originates with the promotion of S_0_→S_2_ (π^1^ π*^1^) which can allow the system enough flexibility to distort the dihedral once the π bond strength has a formal bond order of zero. However, due to orbital polarisation there remains a marginally bonding interaction and some thermal energy is still required to break it. This barrier is computed to be 36.0 kJ mol^−1^ in the case of AZO1 and 27.4 kcal mol^−1^ for AZO2 and takes place at ∡(CNNC) = 135° and ∡(CNNC) = 143° respectively. One metric to measure the π symmetry bond strength from the electronic density is the natural localized molecular orbital (NLMO) bond order taken from the CASCI(4,3) density matrix. A comparative analysis of the azo bond orders in ground (S_0_) and excited (S_2_) states is displayed on Table 8. It may be seen that the calculated π bond order is identical from the outset (S_0_). The S_0_→S_2_ excitation suppresses the π bond strength differently in AZO1 and AZO2. In AZO1, the π bond remnant is stronger than in AZO2 thereby justifying the additional energetic requirement for the dihedral rotation and rendering AZO1 kinetically more inert in the S_2_ state.

Disregarding thermal contributions to the transition state energies, i.e., ΔE^‡^(S_2_) ≈ ΔG^‡^(S_2_) and assuming a high quantum yield for the S_2_ excitation, the Eyring equation yields a k_i_(AZO1)/k_i_(AZO2) ratio of 3.1 × 10^−2^ using the above calculated values which is reasonably close to experimental one (4.8 × 10^−2^). Overall, a 9 kJ mol^−1^ energy difference leads to a decrease in the reaction rate constant close to two orders of magnitude. 

Towards more acute angles one hallmark feature [57] of azobenzene derivatives comes to the fore, which is the conical intersection (CI) allowing S_2_ to configurationally admix with S_0_ leading to the cis isomer, in the region 80° < ∡(–CNNC–) < 100° as the lone pairs (n) become degenerate with the π orbitals. The energy gap in this nonadiabatic coupling ($H_{02}$) is 98.3 kJ mol^−1^ in AZO1 and 83.1 kJ mol^−1^ in AZO2. From the Landau–Zener relation [58], it is known that the probability of transition between the two adiabatic surfaces S_0_ and S_2_ can be given by $e^{-H_{02}^{2}/\alpha }$ where *α* is a collection of constants incorporating nuclear velocity, and the difference in reaction coordinate slope in each adiabatic state. $H_{02}^{2}$ is the squared energy gap between the two states at the avoided crossing. As the values of $H_{02}$ are considerable for both molecules (98.3 kJ mol^−1^ in AZO1 and 83.1 kJ mol^−1^ in AZO2), the probability of radiationless transition is practically zero. Thus, the S_0_←S_2_ transition will require the emission of one photon at 1217 nm (AZO1) and 1437 nm (AZO2) wavelengths (infra-red region).

The potential energy landscapes also afford an overview of the thermal back-isomerization which has the values +74.9 and +74.4 kJ mol^−1^. These values are in good agreement with the relaxation activation energies determined experimentally even neglecting the necessary thermal corrections (zero point, vibrational, rotational and translational). 

In light of the current data, the presence of the –OH group in AZO1 cannot be considered to play a direct role in the rapid back isomerization of AZO1 if one considers this neutral tautomer alone as alluded to in the literature [50]. In view of the acidic properties of AZO1, all the possible tautomeric zwitterionic isomers were optimized at the PBE0/TZP level and their optical properties assessed (see Supporting Information section). 

**Scheme 2:**
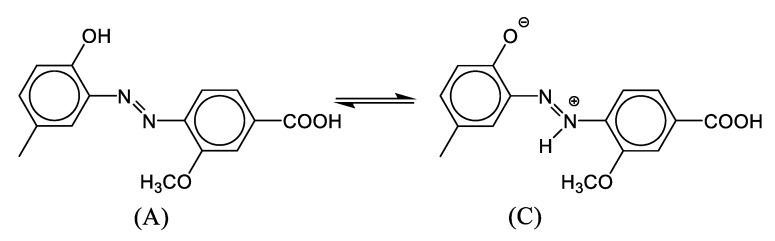
Tautomeric equilibrium between two structural isomers (**A**,**C**) of AZO1.

Our results show that a low energy tautomer may play a role in the isomerization process if the following equilibrium occurs (Scheme 2). This zwitterionic isomer should be more favored in a polar solvent medium [ΔE(DMF) = 3.6 kcal mol^−1^]:

Our results show that the presence of the hydroxy group does indeed influence the conversion process in AZO1, but not by creating strong hydrogen bonds with nitrogen but by instead allowing for the existence of diazo-phenolate that has distinctly unique photodynamic properties (Supplementary Figure S6). 

## 4. Conclusions

In this work, two novel asymmetrical azobenzene derivatives were successfully synthesized, characterized, and studied. A molecular design was done in order to plan and create moieties with one reactive suitable site for polymer functionalization. The molecules were set to have electron-donor groups that permit isomerization when irradiated by visible light irradiation and a sufficiently stable *Z*-isomer with a thermal relaxation at temperatures above 303K. A wide-ranging study on the *E–Z–E* isomerization mechanism of the azo-derivatives was performed. The definite substitutional pattern was found to change the specific photochemistry of the molecules. A hindered isomerization was discovered in the case of AZO1 molecule, subsequently analyzed by the TD-DFT calculations. 

On the other hand, by methylating AZO1 to AZO2, the photochemistry of AZO2 was found to follow the bibliography trends. AZO2 isomerization occurs under visible light irradiation, giving a stable *Z*-isomer at room temperature, with thermal relaxation rate constants increasing at higher temperature, as expected. The interaction between solvent and chromophore plays a role in determining the rate of this process and therefore should be considered in future applications.

The rate of photo-switching was experimentally determined to be k_i_(AZO1) = 1.5 × 10^−4^ s^−1^ and k_i_(AZO2) = 3.08 × 10^−3^ s^−1^ using DMF as solvent.

By means of TD-DFT calculations, the molecules were studied and compared. The calculated bands (TD-PBE0) relate well to the experimental ones, presenting a minor systematic redshift. The conventional assignment of distinctly separated n→ π* and π→ π* transitions was found to be not rigorously valid in the case of *E*-AZO1 since an admixture of both transitions in the first two bands was found. The spectral features of *E*-AZO2 are different in that n→ π* transitions do not admix with the π→ π* type excitations. 

The dynamic photochemical and thermal processes involved in the isomerisation of AZO1 and AZO2 was studied by means of multi-configurational self-consistent field theory with a posteriori perturbational corrections. The rotation mechanism of the planes that make up the ∡(C–N=N–C) dihedral angle was found to be the most favourable for the E-Z isomer change. The photo-chemical process originates with the electronic promotion of S_0_→S_2_ and an energetic barrier in the S_2_ surface was found along the reaction coordinate valued at +36.0 and +27.4 kJ mol^−1^ for AZO1 and AZO2 respectively. This difference in thermal barriers for the S_2_ state explains why AZO1 is kinetically more inert to isomerisation with respect to AZO2. Another route for AZO1 conversion is through dihedral rotation of the proton tautomer but the ground state of the Z isomer in this instance is thermodynamically unstable and rapidly switches back to the E isomer. This is consistent with the spectral evidence.

While the topology of the hydrogen bonds in the ortho position may be relevant in the photo-switching of certain azo derivatives, this feature is entirely reliant on the nature of the most favourable reaction coordinate. In light of previous results [53], this shows that subtle interplays between substituent groups and the aryl rings pose difficulties in the modular design of tuneable azo compounds since their interconversion shows up as system specific. 

The photo-switching in AZO1 is inhibited due to a stronger π bond order hindering rotation around the N–N bond axis. Further studies will be required to attempt to draw systematic functional group trends within the same mechanistic scenario. 

To conclude, in this work a molecular design approach was used to plan and produce two novel moieties, suitable candidates for applications that aim to take advantage of visible light irradiation. Combined with TD-DFT calculation, a comprehensive study of their properties was achieved giving also more information about the important role of the substituents that must be selected during the planning phase of these molecules. These findings can provide new insights regarding substituted azobenzene isomerization as well as the possibility to reveal new applications for a well-known organic molecule.

## Figures and Tables

**Table 1:** ^13^C NMR chemical shifts comparison of AZO1 and AZO2 molecules.

	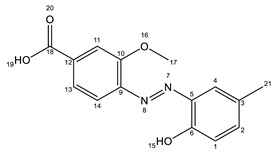 AZO1	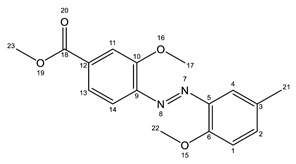 AZO2
1	118.2	113.3
2	135.1	133.0
3	134.2	129.4
4	128.1	113.6
5	141.7	142.2
6	151.7	144.5
9	138.1	141.7
10	155.5	154.8
11	113.7	116.6
12	128.9	156.0
13	122.0	121.4
14	116.1	115.4
17	56.3	55.8
18	166.7	169.1
21	19.9	20.1
22	-	56.1
23	-	55.8

**Table 2:** Wavelength maxima and experimentally measured rate constants for isomerization (k_i_), reverse isomerization (k_r_) and the content of the Z-isomer at the PSS (α_Z_). AZO1 and AZO2 optical properties comparison. * = the obtained value is not reliable because it is falling within the experimental uncertainty of the instrument (see text).

Sample	Solvent	λ_1_ (nm)	λ_2_ (nm)	k_i_ (s^−1^)	k_r_ (s^−1^)	α_Z_ (%)
**AZO1**	DMF	415	330	1.5 × 10^−4^	*	3.2
**AZO2**	DMF	389	320	3.08 × 10^−3^	3.77 × 10^-6^	45.7

**Table 3:** Calculated excitation energies, band maxima assignments and oscillator strengths (*f*_osc_) of *E*-AZO1 (experimental values from UV/Vis analysis are shown in brackets).

Excited State #	Main Character	Excitation (nm)	*f*_osc_
**S_1_**	π_2_→ π* (48%) n_1_→ π* (37%) n_2_→ π* (12%)	489 (415)	0.34
**S_2_**	π_2_→ π* (51%) n_1_→ π* (36%) n_2_→ π* (11%)	441 (399)	0.57
**S_3_**	n_2_→ π* (76%) n_1_→ π* (23%)	379	6.9 × 10^−2^
**S_4_**	π_1_→ π* (98%)	338 (330)	0.33

**Table 4:** Calculated excitation energies, band maxima assignments and oscillator strengths (*f*_osc_) of *E*-AZO2 (experimental values from UV/Vis analysis are shown in brackets).

Excited State #	Main Character	Excitation (nm)	*f*_osc_
**S_1_**	n_2_→ π* (58%) n_1_→ π* (37%)	494	9.6 × 10^−3^
**S_2_**	π_2_→ π* (98%)	448 (389)	0.91
**S_3_**	n_1_→ π* (59%) n_2_→ π* (39%)	372	6.6 × 10^−2^
**S_4_**	π_1_→ π* (98%)	333 (320)	0.30

**Table 5:** AZO2 *E-Z* photoisomerization rate constants at 303K. In this order: k_i_, direct isomerization kinetic constant; k_r_, thermal relaxation kinetic constant; α_Z_, content of the *Z*-isomer at the PSS (photo-stationary state); τ_1/2_, half-life of *Z*-isomer in the dark.

Solvent	k_i_ (s^−1^)	k_r_ (s^−1^)	α_Z_ (%)	τ_1/2_(s)
DMF	3.08 × 10^−3^	3.77 × 10^−6^	45.7	1.84 × 10^5^
DMSO	6.38 × 10^−3^	3.14 × 10^−6^	48.3	2.21 × 10^5^
DIOX	8.51 × 10^−4^	2.14 × 10^−6^	16.7	3.24 × 10^5^

**Table 6:** AZO2 k_r_ (in s^−1^) constants in different solvents at different temperatures.

T (K)	DMF	DMSO	DIOX
303.15	3.77 × 10^−6^	3.14 × 10^−6^	2.14 × 10^−6^
313.15	1.18 × 10^−5^	1.02 × 10^−5^	6.23 × 10^−6^
323.15	3.22 × 10^−5^	3.01 × 10^−5^	1.73 × 10^−5^
333.15	5.59 × 10^−5^	6.93 × 10^−5^	3.81 × 10^−5^

**Table 7:** AZO2 thermodynamic activation parameters.

Solvent	Dipole Moment (D)	A (s^−1^)	E_a_ (kJ mol^−1^)	ΔH^‡^ (kJ mol^−1^)	ΔS^‡^ (J mol^−1^ K^−1^)
DMF	3.86	6.57 × 10^7^	76.6	74.0	−104.09
DMSO	4.1	3.41 × 10^9^	87.2	84.5	−71.25
DIOX	0.45	2.22 × 10^8^	81.3	78.6	−94.03

**Table 8:** NLMO bond orders in the two states S_0_ and S_2_ at the ground state CASCI (4,3)/def2-TZVP level.

	NLMO π(N–N) Bond Order S_0_	NLMO π(N–N) Bond Order S_2_
AZO1	0.951	7.84×10^−3^
AZO2	0.934	4.43×10^−3^

## Supplementary Materials

The following are available online at https://www.mdpi.com/2073-4360/12/5/1019/s1, Figure S1—(A) Emission spectra of white, blue, green, yellow, red lights emitted from the tuneable Jedi Lighting iDual lamp employed as irradiating source, and (B) corresponding illumination values measured at 2 and 8 cm from the light source; Figure S2—QD-NEVPT2//CASSCF(4,3)/def2-TZVP Potential energy surface scans of AZO1 (top) and AZO2 (bottom) relative to the ∡(NNC) angle twisting to the linear position, Figure S3– Tight grid scan of AZO1 (left) and AZO2 (right) evidencing the two (avoided) crossings of S_2_ and S_3_, Table S1—Relative energies of the zwitterionic isomers of AZO1 as calculated through PBE0/TZP (DMF); Table S2—Calculated UV/Vis absorption spectrum of E-AZO1-C at the PBE0/TZP (DMF) level of theory; Figure S4—Calculated UV-Vis spectra of the two tautomers (convoluted with a Gaussian function at FWHM=60); Figure S5—Active space composition of the state average CAS(4,3)SCF wavefunction of E-AZO1-C; Table S3—Active space composition of E-AZO1-C (first point in PES of Figure S3 below); Figure S6—Potential energy surfaces (QD-NEVPT2/def2-TZVP) with respect to dihedral rotation in AZO1-C; Figure S7—QD-NEVPT2//CASSCF(4,3)/def2-TZVP Potential energy surface scans of AZO1-C relative to the R (NNC) angle twisting to the linear position; Table S4—Calculated band absorption wavelengths for the AZO1 system; Figure S8—^1^H NMR AZO2 spectrum at PPS in d-DMSO solution at 30 °C; Figure S9—First-order kinetic of the Z-E thermal relaxation of AZO2 in DMF solution at 30 °C; Figure S10—Tentative fitting of AZO1 thermal relaxation kinetic constant in DMF solution at 30 °C.

## Appendix A

### Appendix A.1. Theory and Calculations

The photoisomerization experimental data were analyzed according to Equation (A1).

(A1) $$\ln\frac{A_{0}-A_{\infty }}{A_{t}-A_{\infty }}=k_{i}t$$

where *t* is the irradiation time, *A_0_*, *A_t_* and *A_∞_* are the *E* form absorbances corresponding to the time *0*, *t* and photostationary state (PSS), respectively, and *k_i_* is the rate constant of *E–Z* photoisomerization. In the case of thermal relaxation, the kinetics follow Equation (A2), where *k_r_* is the rate constant of the *Z–E* relaxation, *A_0_*, *A_t_* and *A_∞_* are the *Z* form absorbances corresponding to the time *0*, *t* and photostationary state and *t* is the relaxation time.

(A2) $$\ln\frac{A_{0}-A_{\infty }}{A_{t}-A_{\infty }}=k_{r}t$$

The content of the *Z*-isomer at the PSS was determined using Equation (A3).

(A3) $$\alpha_{Z}=\frac{A_{0}-A_{PSS}}{A_{0}}$$

where *A_0_* and *A_PSS_* represent the absorbances at *λ_max_* for *E* isomer before and after irradiation with UV light, respectively. Activation parameters of the thermal back isomerization were determined by measuring the temperature dependence of the rate constant and fitting the data with the Arrhenius equation (Equation (A4)) or the Eyring equation (Equation (A5)) to obtain the activation energy, *E_a_*, the enthalpy of activation, Δ*H^‡^*, and the entropy of activation, Δ*S^‡^*.

(A4) $$\ln k=\ln A-\frac{E_{a}}{RT}$$

where *k* is the thermal rate constant, *R* is the universal gas constant, *T* is the temperature in Kelvin, *A* is the steric factor and *E_a_* is the activation energy.

(A5) $$\ln\frac{k}{T}=-\frac{\Delta H^{‡}}{RT}+\ln\frac{k_{b}}{h}+\frac{\Delta S^{‡}}{R}$$

where Δ*H*^‡^ is the enthalpy of activation, Δ*S*^‡^ the entropy of activation, *k_b_* is the Boltzmann constant, *h* is Planck constant.
