# Supplementary material for: Contrasting Photo-Switching Rates in Azobenzene Derivatives: How the Nature of the Substituent Plays a Role
Source: Polymers (Basel). 2020 Apr 30;12(5):1019. doi: 10.3390/polym12051019 (PMC7284787; doi:10.3390/polym12051019)
Supplement: Supplementary file 1 [file polymers-12-01019-s001.pdf]

Supporting Document section

1. iDual lamp spectrum:

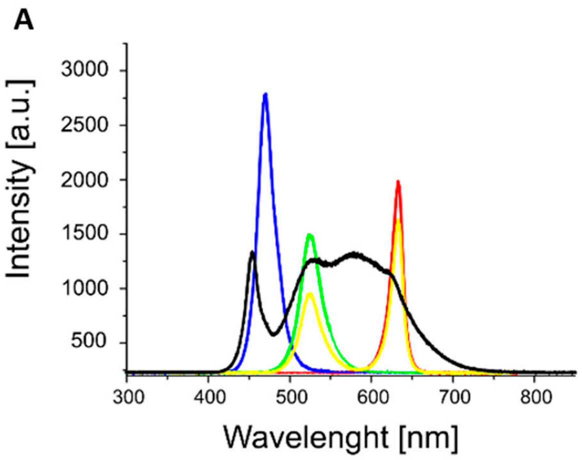

B

Light source	Illumination (lux) (2 cm)	Illumination (lux) (8 cm)
White	3890	1030
Blue	2170	552
Green	1640	383
Yellow	1540	374
Red	1028	198

Figure S1 - (A) Emission spectra of white, blue, green, yellow, red lights emitted from the tuneable Jedi Lighting iDual lamp employed as irradiating source, and (B) corresponding illumination values measured at 2 and 8 cm from the light source.

2. Twisting mechanism

A potential energy scan was performed for both AZO1 and AZO2 compounds for the twisting of the ∡CNN angle starting from the trans equilibrium geometry and ending in the linear *sp* hybridized transition state. Since both aromatic rings have different substituents a twisting of both sites was performed. These results are displayed on Figure S2. Clearly the S2 pathway has a considerable thermal barrier (close to 20 kcal mol^{-1}) in the case of AZO1. This is much lower in the case of AZO2 where the value drops by almost half. The magnitude of this barrier is likely related to the steric repulsion caused by the in plane lone pair of nitrogen which must adapt to the linearization, i.e.,

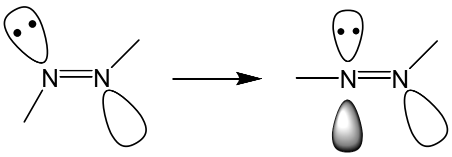

(Scheme 1)

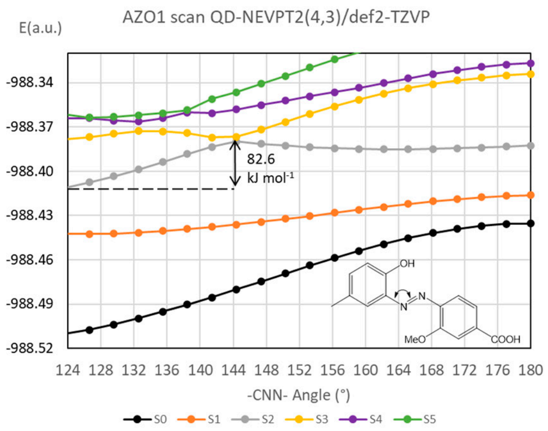

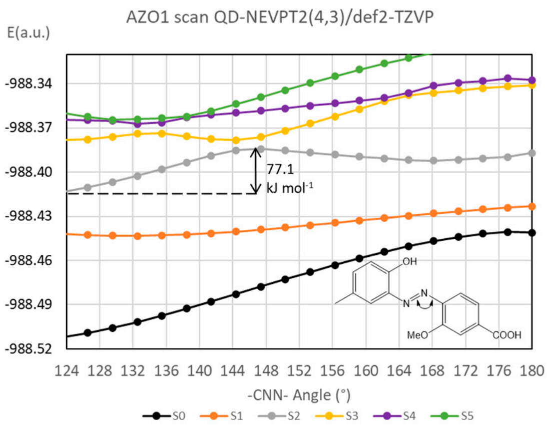

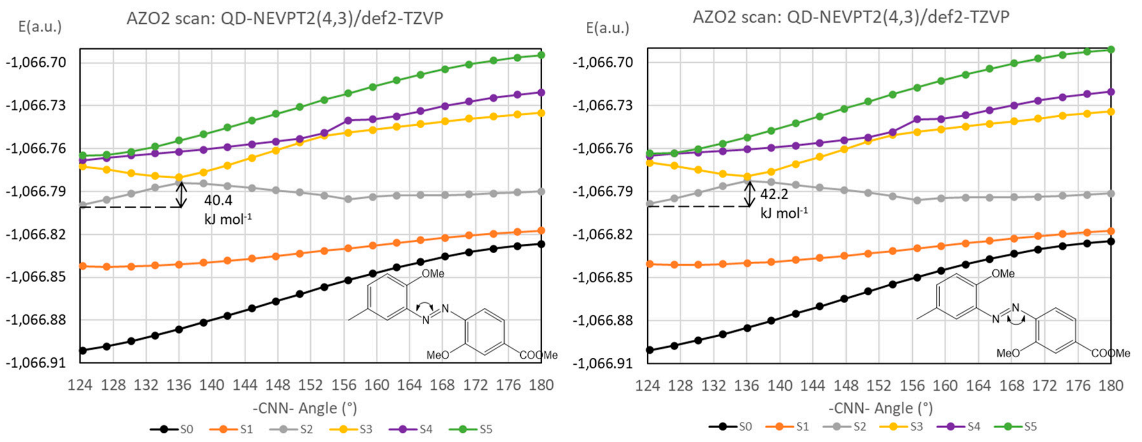

Figure S2 – QD-NEVPT2//CASSCF(4,3)/def2-TZVP Potential energy surface scans of AZO1 (top) and AZO2(bottom) relative to the ∡(NNC) angle twisting to the linear position.

If one compares the NEVPT2 Mulliken reduced populations of the 2p orbitals in nitrogen, it may be seen that q(AZO1) = 3.6 and q(AZO2) = 3.5 while the 2s populations remain steady at 1.5 in both compounds (at their respective equilibrium position). For this reason, the higher charge in AZO1 is likely the reason why the thermal barrier is higher for this compound since a larger repulsion has to be overcome.

The thermal back-isomerization starting from the Z isomer is evaluated to be $\Delta E^{\ddagger}$=28.4 kcal mol^{-1} for AZO1 and 32.6 kcal mol^{-1} for AZO2. These are comparably larger than the values reported in the main text for the rotation mechanism (17.9 and 14.0 kcal mol^{-1}).

3. Avoided crossings

As support for Figures 7–8, a tighter grid of potential energy points was carried out near the crossing regions between S2 and S3. While it is clear that AZO1 has an avoided crossing with a 11.3 kJ mol^{-1} energy gap, AZO2 has a state crossing which cannot be clearly resolved at this level of theory as the points differ by an order of magnitude less than the accuracy of the method itself 0.8 kJ mol^{-1} (Supplementary Figure S. This may be a very weakly avoided crossing, but it cannot be confirmed with certainty.

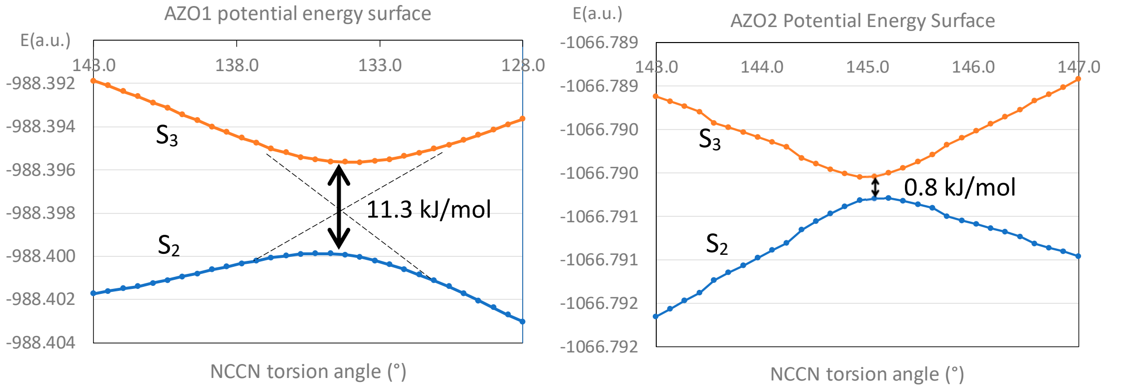

Figure S3 – Tight grid scan of AZO1 (left) and AZO2 (right) evidencing the two (avoided) crossings of S_2 and S_3.

4. Proton tautomers

A geometry optimisation was performed on the several proton tautomers of AZO1 to establish if any protonation equilibria might play a role in the photo-conversion. Only structure C is shown to have an energetically accessible value (3.6 kcal mol^{-1}), but there is no obvious coupled proton transfer during the photochemical process as the protonated nitrogen is distant from the phenolate group.

Table S1 – Relative energies of the zwitterionic isomers of AZO1 as calculated through PBE0/TZP (DMF).

trans-AZO1 Isomer	ΔE(kcal.mol^{-1})
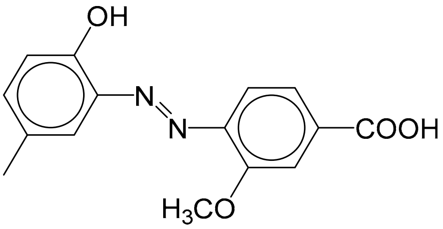 (A)	0
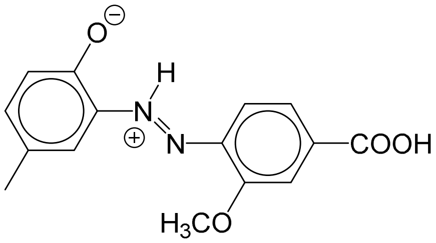 (B)	+7.5
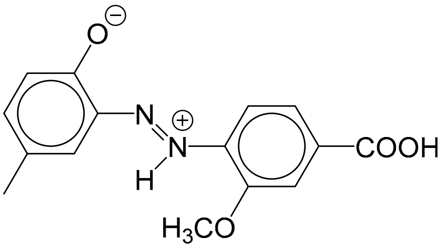 (C)	+3.6
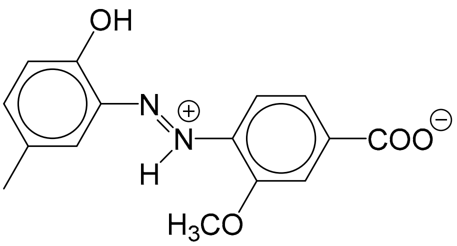 (D)	+9.4
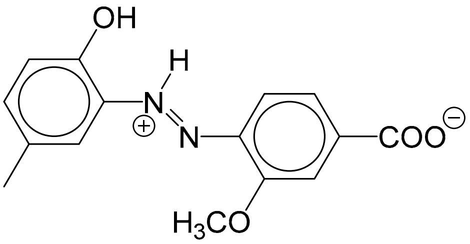 (E)	+12.5

As trans-AZO1-C presents an energetically competitive energy with respect to trans-AZO1-A, TDDFT calculations were also performed on the former. The result is that the most efficient excitation channel in the visible region is through S_1 (Supplementary Table S2) due to the considerably high value of its oscillator strength.

Table S2 – Calculated UV/Vis absorption spectrum of E-AZO1-C at the PBE0/TZP (DMF) level of theory.

State	λ(nm)	f_{osc}
**S_1**	545 (red)	0.874
**S_2**	420	1.81×10^{-4}
**S_3**	393	0.0634
**S_4**	343	0.162
**S_5**	306	0.241

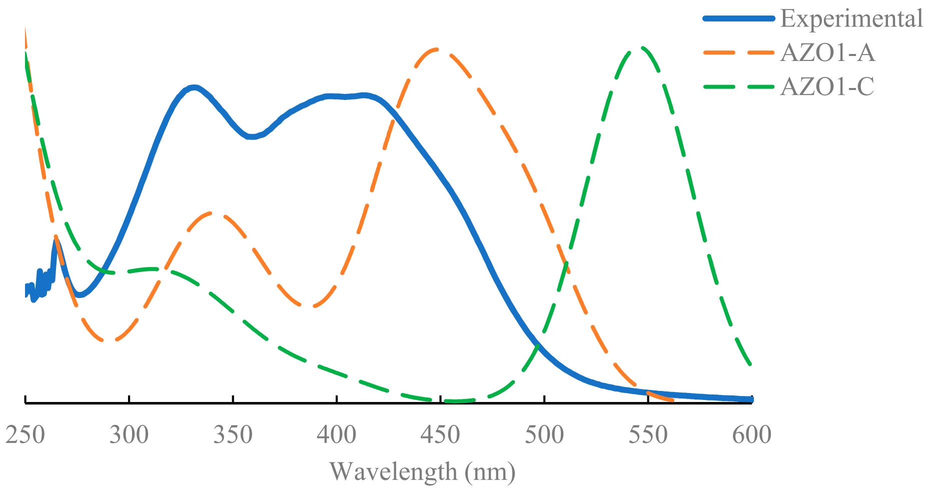

Figure S4 – Calculated UV-Vis spectra of the two tautomers (convoluted with a Gaussian function at FWHM=60).

Wavefunction calculations were also performed on the AZO1-C species and the results are summarized below.

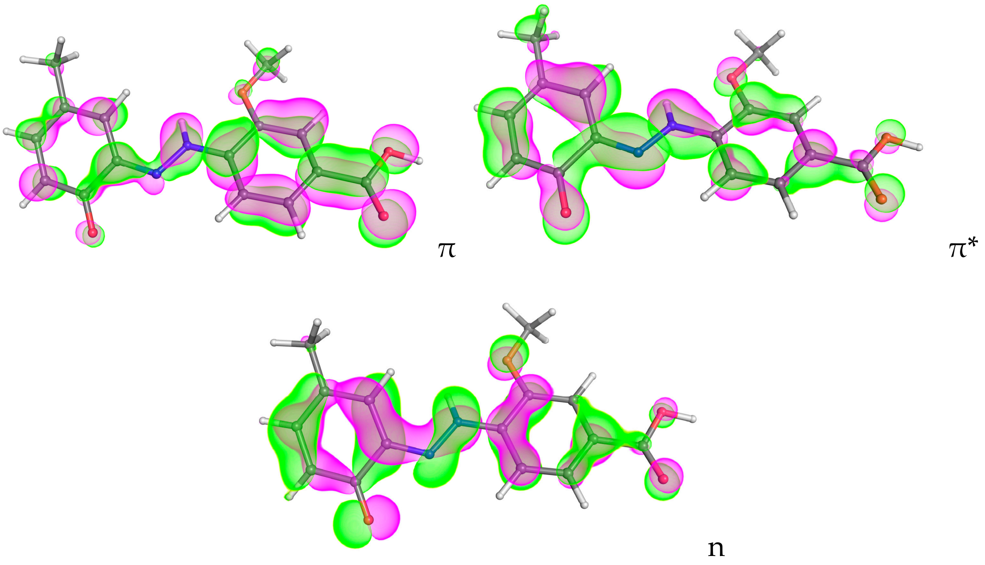

Figure S 5 – Active space composition of the state average CAS(4,3)SCF wavefunction of E-AZO1-C.

Table S 3 – Active space composition of E-AZO1-C (first point in PES of Figure S3 below).

States	Leading Configurations of QD-NEVPT2 density	E(cm^{-1})
S_0	54%|$n^2\ \pi^1\ \pi^{*1}$><br>41% |$n^2\ \pi^0\ \pi^{*2}$>	0
S_1	71% |$n^2\ \pi^2\ \pi^{*0}$><br>11% |$n^2\ \pi^0\ \pi^{*2}$>	27542
S_2	67% |$n^1\ \pi^1\ \pi^{*2}$><br>15%|$n^1\ \pi^2\ \pi^{*1}$><br>13% |$n^2\ \pi^2\ \pi^{*0}$>	29813
S_3	36%|$n^1\ \pi^2\ \pi^{*1}$><br>32%|$n^2\ \pi^0\ \pi^{*2}$><br>23%|$n^2\ \pi^1\ \pi^{*1}$>	44990
S_4	40% |$n^1\ \pi^2\ \pi^{*1}$><br>20% |$n^1\ \pi^1\ \pi^{*2}$><br>14% |$n^2\ \pi^0\ \pi^{*2}$><br>13% |$n^2\ \pi^1\ \pi^{*1}$>	60353
S_5	88% |$n^0\ \pi^2\ \pi^{*2}$>	70885

A dynamic analysis of the dihedral rotation was also performed for AZO1-C (Supplementary Figure S6, below).

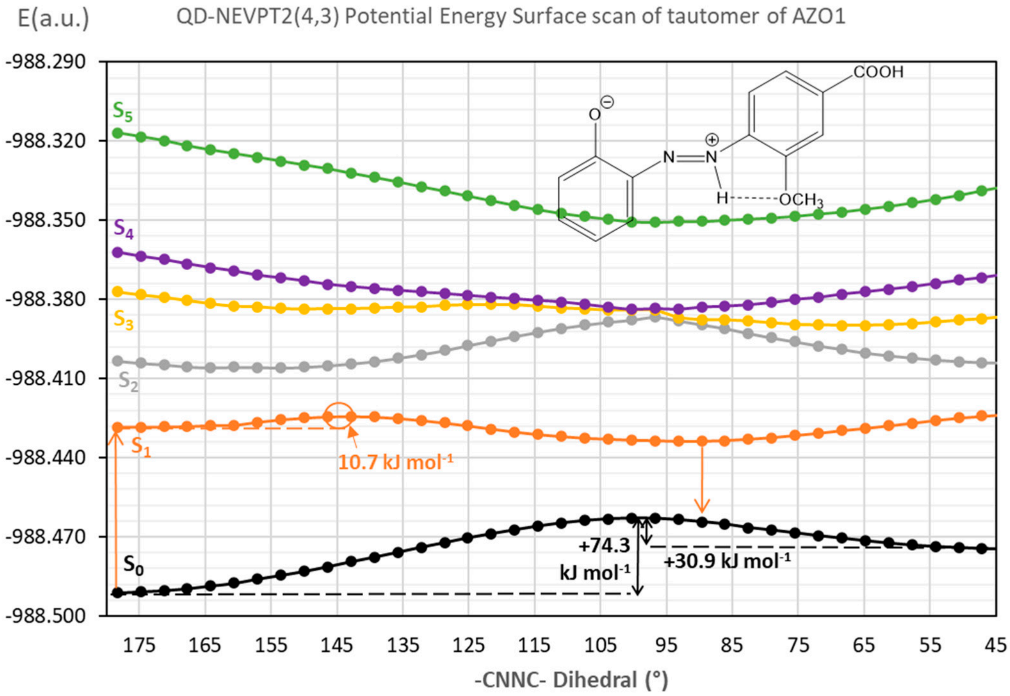

Figure S 6 – Potential energy surfaces (QD-NEVPT2/def2-TZVP) with respect to dihedral rotation in AZO1-C.

The first excited state (S_1) exhibits a flat potential energy surface which would be consistent with a rapid rotation and subsequent de-excitation onto S_0. The Z isomer

molecule is thermally labile with a very low kinetic barrier for back-isomerisation (30.9 kJ mol^{-1}, 7.4 kcal mol^{-1}) as well as being thermodynamically unstable (+43.4 kJ mol^{-1}) with respect to the E isomer.

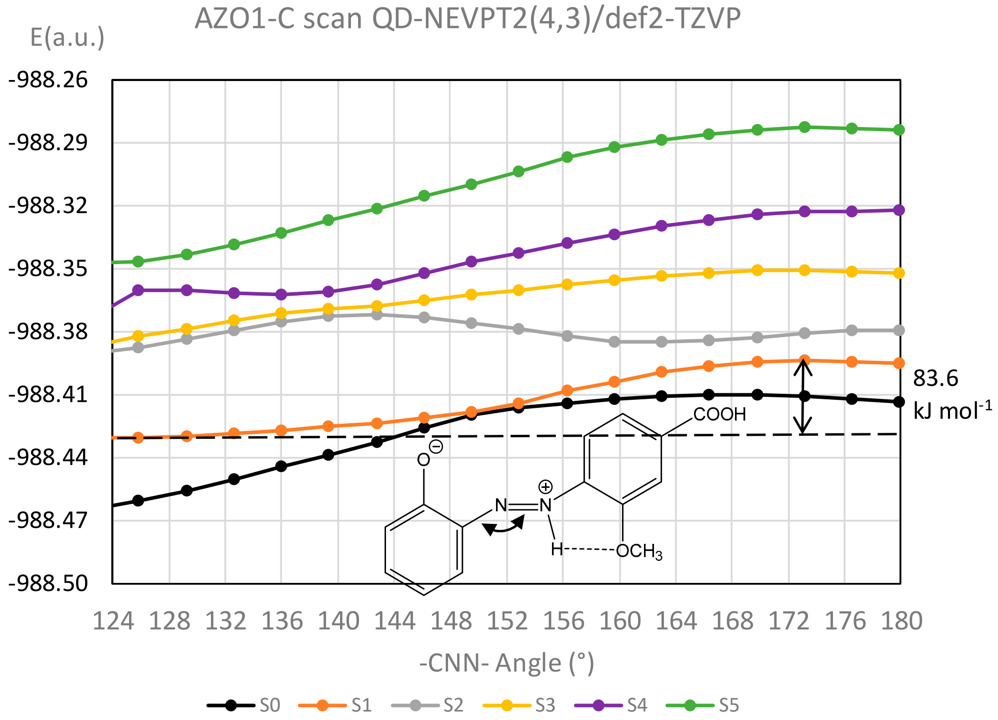

Figure S 7 - QD-NEVPT2//CASSCF(4,3)/def2-TZVP Potential energy surface scans of AZO1-C relative to the ∡(NNC) angle twisting to the linear position.

5. UV/Vis discussion:

The *E*-AZO1 UV-Vis spectrum was calculated in various solvents to determine how their polarity can shift the visible band wavelengths.

Table S4 - Calculated band absorption wavelengths for the AZO1 system.

COSMO dielectric medium	λ_1(nm)	λ_3(nm)
DMSO	449	340
DMF	441	338
CH_2Cl_2	444	338
None	410	322

6. ^{1}H NMR AZO2 spectrum at PPS in d-DMSO solution at 30 °C:

In Supplementary Figure S7, the comparison between the ^{1}H NMR spectra of AZO2 sample in d-DMSO without irradiation (black line) and the same sample at PPS (red line) after white light irradiation at 30 °C is shown.

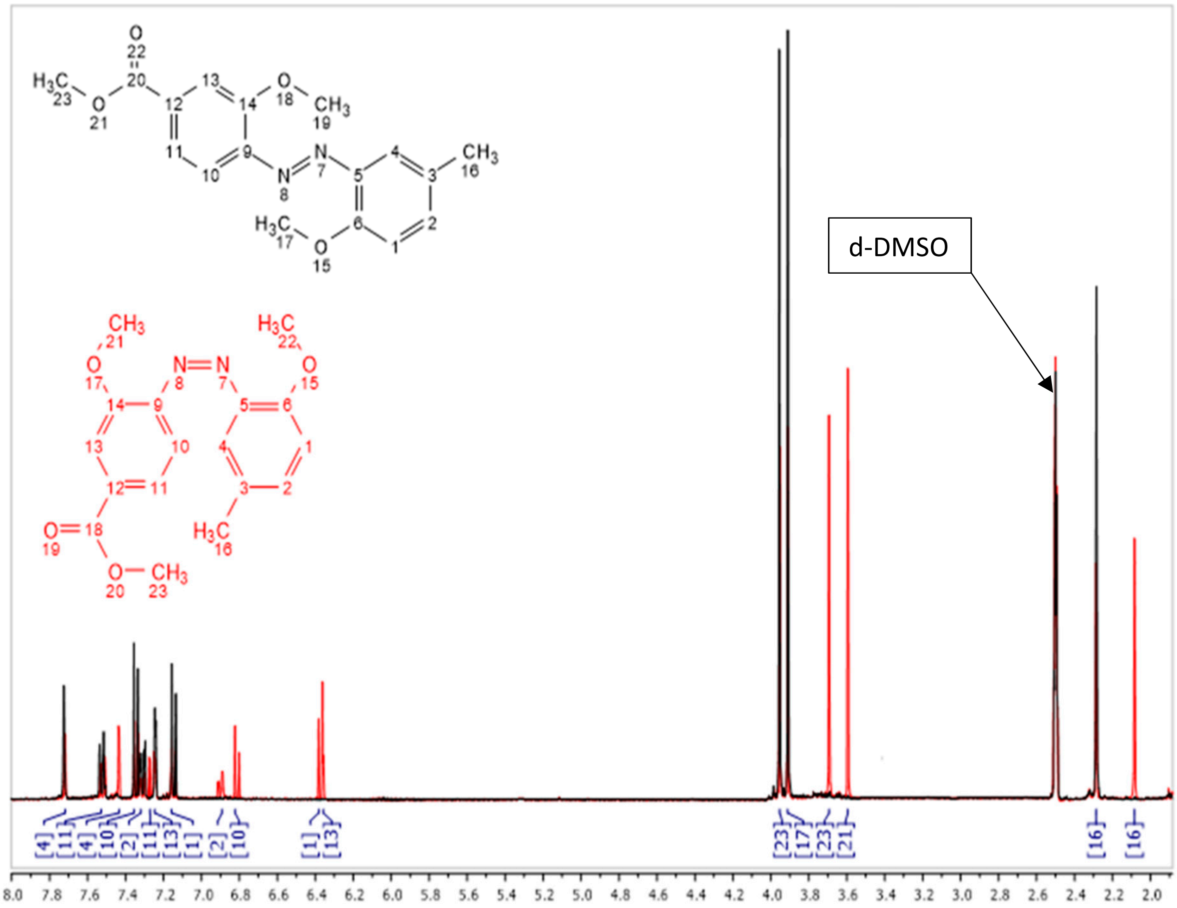

Figure S 8 - ¹H NMR AZO2 spectrum at PPS in d-DMSO solution at 30 °C.

7. Kinetic data of AZO2 thermal relaxation:

AZO2 kinetic data of the thermal relaxation mechanism in DMF at 30 °C.

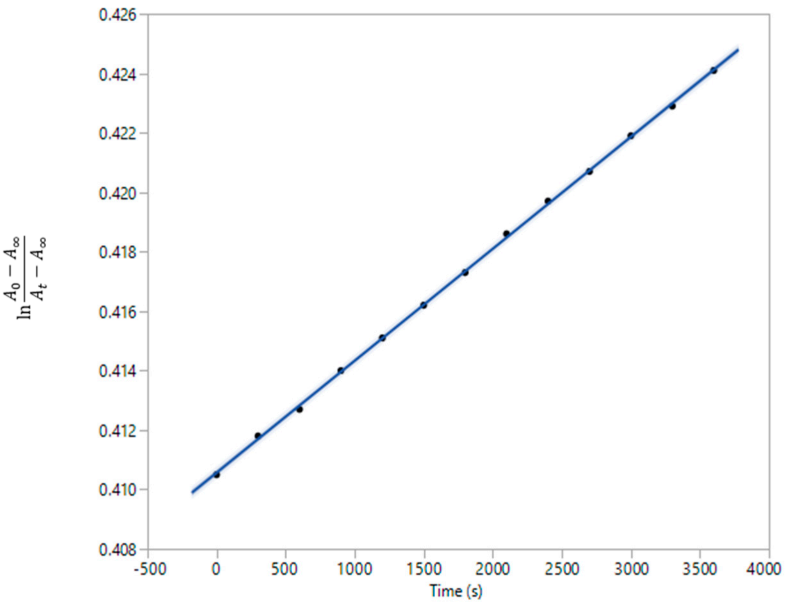

Figure S 9 - First-order kinetics of the Z-E thermal relaxation of AZO2 in DMF solution at 30 °C.

8. Tentative fitting of AZO1 thermal relaxation kinetic constant:

A rough estimation of the kinetic constant relative to *Z-E* thermal relaxation of AZO1 was attempted from the data obtained in DMF.

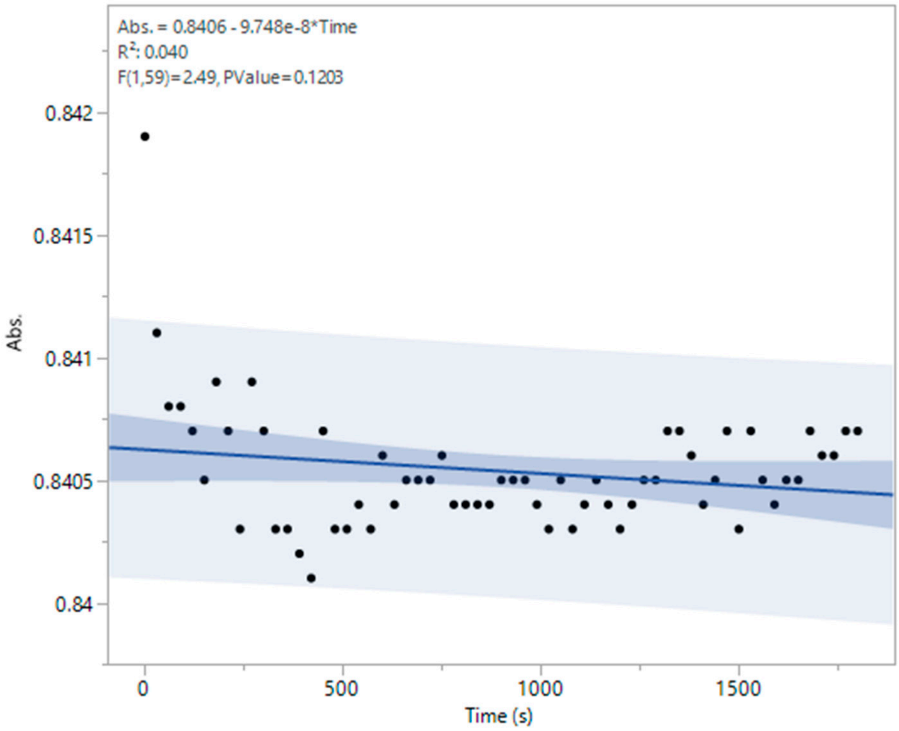

Figure S 10 - Tentative fitting of AZO1 thermal relaxation kinetic constant in DMF solution at 30 °C.
